# *Caesalpinia sappan* Linn. Ameliorates Allergic Nasal Inflammation by Upregulating the Keap1/Nrf2/HO-1 Pathway in an Allergic Rhinitis Mouse Model and Nasal Epithelial Cells

**DOI:** 10.3390/antiox11112256

**Published:** 2022-11-15

**Authors:** Bo-Jeong Pyun, Kyuhyung Jo, Joo Young Lee, Ami Lee, Myung-A Jung, Youn-Hwan Hwang, Dong Ho Jung, Kon-Young Ji, Susanna Choi, Yun Hee Kim, Taesoo Kim

**Affiliations:** KM Convergence Research Division, Korea Institute of Oriental Medicine, Daejeon 34054, Republic of Korea

**Keywords:** *Caesalpinia sappan* Linn., allergic rhinitis, Th2 cytokine, ROS, inflammation, antioxidant, Keap1/Nrf2/HO-1 signaling pathway

## Abstract

Allergic rhinitis (AR) is a common upper-airway inflammatory disease of the nasal mucosa caused by immunoglobulin (IgE)-mediated inflammation. AR causes various painful clinical symptoms of the nasal mucosa that worsen the quality of daily life, necessitating the urgent development of therapeutic agents. Herein, we investigated the effects of *Caesalpinia sappan* Linn. heartwood water extract (CSLW), which has anti-inflammatory and antioxidant properties, on AR-related inflammatory responses. We examined the anti-inflammatory and anti-allergic effects of CSLW in ovalbumin (OVA)-induced AR mice and in primary human nasal epithelial cells (HNEpCs). Administration of CSLW mitigated allergic nasal symptoms in AR mice, decreased total immune cell and eosinophil counts in nasal lavage fluid, and significantly reduced serum levels of OVA-specific IgE, histamine, and Th2 inflammation-related cytokines. CSLW also inhibited the infiltration of several inflammatory and goblet cells, thereby ameliorating OVA-induced thickening of the nasal mucosa tissue. We found that CSLW treatment significantly reduced infiltration of eosinophils and production of periostin, MUC5AC, and intracellular reactive oxygen species through the Keap1/Nrf2/HO-1 pathway in HNEpCs. Thus, our findings strongly indicate that CSLW is a potent therapeutic agent for AR and can improve the daily life of patients by controlling the allergic inflammatory reaction of the nasal epithelium.

## 1. Introduction

Allergic rhinitis (AR) is a chronic upper respiratory disorder characterized by inflammation of the nasal mucosa that affects approximately 10–40% of the global population [1,2]. AR is caused by immunoglobulin (Ig) E-mediated reactions to specific inhaled allergens and is not a life-threatening disorder, but it can worsen the quality of life by causing clinical symptoms such as sneezing, nasal itching, rhinorrhea (nasal discharge), and congestion [1,3,4]. These inflammatory reactions are associated with the infiltration and activation of several immune cells, such as mast cells, basophils, and eosinophils, which are controlled by T helper type 2 (Th2) cells and their cytokines [3,5].

In respiratory diseases, Th2 inflammatory cytokines (interleukin [IL]-4, IL-5, and IL-13) increase the expression of periostin and eotaxins, which are major mediators of eosinophilic inflammation and airway remodeling [6,7,8]. Periostin is a matricellular protein that is highly expressed in several types of cells in most illness conditions, including chronic airway disease, and it has multiple functions, including tissue regeneration and the remodeling (such as fibrosis and collagen deposition) of inflammatory diseases [8,9]. Eotaxin-3 (also known as CCL26) is a chemoattractant for eosinophils and basophils and promotes the migration and accumulation of eosinophils to inflammatory sites in the nasal epithelium [8,10]. Additionally, cells in the upper respiratory epithelium, such as nasal epithelial cells, produce regulators that increase mucus-related gene expression and mucus secretion. Mucin 5 subtype AC (*MUC5AC*), a secretory mucin gene, is known to regulate mucus production in the airways [11,12]. Mucus is secreted by goblet cells, which are specialized secretory epithelial cells that line the mucosal surfaces of the airways [13,14]. In chronic inflammatory conditions, goblet cell hyperplasia and MUC5AC overproduction due to epithelium damage result in hypersecretion of mucus, leading to mucociliary dysfunction that eventually aggravates AR symptoms [12,15]. Ultimately, the airway epithelium plays a major role in the inflammatory reactions that induce AR symptoms, with allergen exposure of the nasal mucosa serving as the initiation point of allergic responses [1,16]. Therefore, the regulation of several key immune cells and allergic mediators involved in inflammatory responses in the respiratory tract epithelium may represent an influential therapeutic strategy for alleviating inflammation in AR [16,17].

Oxidative stress caused by imbalances between oxidative power and antioxidant defense mechanisms leads to various physiological and pathological conditions and is directly related to chronic inflammation [5,18]. During AR pathogenesis, reactive oxygen species (ROS) are produced excessively, suppressing antioxidant system function and promoting inflammatory response [18,19]. Nuclear factor erythroid 2-related factor 2 (Nrf2) is a redox-sensitive transcription factor [20]. The activation of Nrf2 signaling is regulated by Kelch-like ECH-associated protein 1 (Keap1), which segregates Nrf2 from the cytoplasm or promotes Nrf2 ubiquitination and degradation [21,22]. Under oxidative stress conditions, Nrf2 is separated from Keap1 and translocated into the nucleus, where it binds to the antioxidant response element and induces the expression of antioxidant enzymes such as heme oxygenase-1 (HO-1), NAD(P)H, quinone oxidoreductase (NQO1), and superoxide dismutase (SOD) [22,23]. These antioxidant enzymes promote ROS detoxification and serve as biomarkers of oxidative stress [24,25]. To treat AR, it is also necessary to regulate antioxidant activity and ROS production.

*Caesalpinia sappan* Linn. (*C. sappan*), known as Brazil redwood or Sappan wood, is a traditional medicinal plant distributed in South India and Southeast Asia, including Sri Lanka, Vietnam, and China [26,27,28]. The dried heartwood of *C. sappan* is commonly used as a natural red dye and material for food or beverages, and it has been reported to possess several pharmacological characteristics, including antioxidant, anti-inflammatory, anti-hyperglycemic, anti-hypercholesterolemic, anti-allergic, anti-arthritic, analgesic, and vasorelaxant activities [26,27,29]. *C. sappan* heartwood mainly comprises phenolic compounds such as brazilin, protosappanins, chalcones, and homoisoflavones [27,30]. Brazilin, the major active compound of *C. sappan* heartwood, is used as a natural red pigment for histological staining [30] and it exhibits almost all of the medicinal properties associated with *C. sappan* heartwood [30,31]. However, few studies have investigated the anti-allergic effect of *C. sappan* heartwood and the mechanisms of allergen-induced nasal epithelial inflammatory responses.

In the present study, we investigated the effect of water-extracted *C. sappan* heartwood (CSLW) on the pathophysiological properties of nasal mucosa in ovalbumin (OVA)-induced AR mice. We also investigated its effects on the underlying mechanisms of ROS production and Keap1/Nrf2/HO-1 pathway regulation to examine the relationship between Th2-related inflammatory mediators (eotaxin-3, periostin, MUC5AC) and allergic inflammation, using IL-4- and IL-13 (IL-4/IL-13)-induced primary human nasal epithelial cells (HNEpCs). In short, we focused on the Th2-immunomodulatory and antioxidant effects of CSLW in an AR mouse model and in primary HNEpCs. This is the first study to highlight the potential of CSLW for AR treatment.

## 2. Materials and Methods

### 2.1. Preparation and Chemical Profiling of CSLW

**Figure 1:**
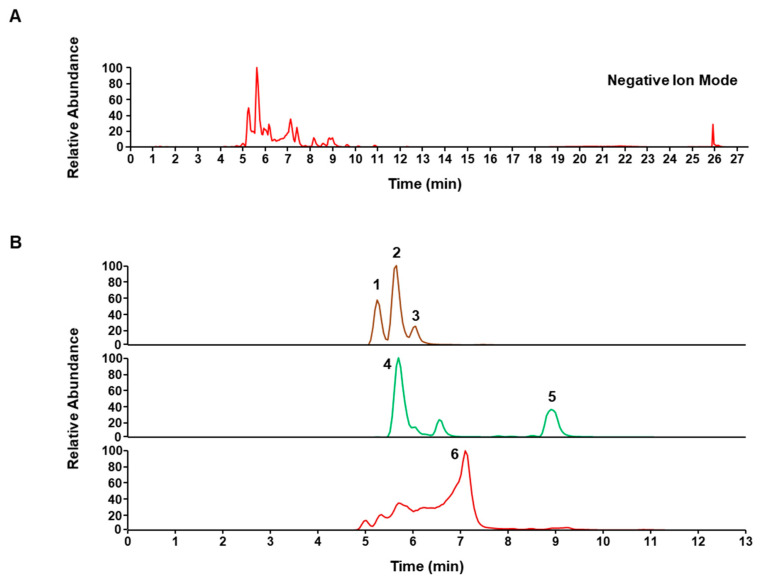
UPLC-MS/MS analysis of CSLW. (**A**) Total ion chromatogram in negative ion mode of CSLW. (**B**) Extracted ion chromatograms of CSLW. 1, Episappanol; 2, Protosappanin B; 3, Sappanol; 4, Brazilin; 5, 3-deoxysappanone B; 6, Brazilein. Identified compound **2** was compared with the mass spectrum and retention time of the reference standards.

*C. sappan* heartwood was purchased from Ungok Herb Co. (Busan, Korea). The voucher specimen (voucher number #JW-102) and herbal component were stored in the herbarium of the KM Convergence Research of the Korea Institute of Oriental Medicine (KIOM, Daejeon, Korea). Air-dried *C. sappan* heartwood (1kg) was refluxed with distilled water (10L, CSLW) at 100 ± 2 °C for 3 h, the extract was filtered through a 53 μm mesh filter, concentrated using a rotary evaporator, and finally dried using a lyophilizer. Freeze-dried CSLW powder was stored at −20 °C until further use. To identify the major components of CSLW, chromatographic analysis was performed using a Dionex UltiMate 3000 system, coupled with a Thermo Q-Exactive mass spectrometer (UPLC–MS/MS, Thermo Fisher Scientific, San Jose, CA, USA), according to previously reported methods [32]. Chromatographic separation was achieved using an Acquity BEH C18 analytical column (100 × 2.1 mm, 1.7 μm) with a mobile phase consisting of 0.1% formic acid in water and acetonitrile. Data were acquired and analyzed using Xcalibur and TraceFinder 5.1 software (Thermo Fisher Scientific). As shown in Figure 1 and Table 1, episappanol (1), protosappanin B (2), sappanol (3), brazilin (4), 3-deoxysappanone B (5), and brazilein (6) were identified in CSLW. The retention time and mass spectra of protosappanin B were compared with reference standards (Targetmol, Wellesley Hills, MA, USA); five phytochemicals are consistent with those of previous studies [27,33,34,35].

### 2.2. Animals

Female BALB/c mice (six weeks old, 18–20 g) were purchased from Samtako Inc. (Osan, South Korea). The mice were housed under standard conditions (temperature 22 °C ± 2 °C, humidity 55% ± 15%, 12 h night/12 h day cycle) and were provided ad libitum access to water and food during the experiment. All animal studies were performed in accordance with the Care and Use of Experimental Animals and were approved by the Chonnam National University Institutional Animal Care and Use Committee (approval number CNU IACUC-YB-2021-104).

### 2.3. OVA-Induced AR Mouse Model and Treatment

**Figure 2:**
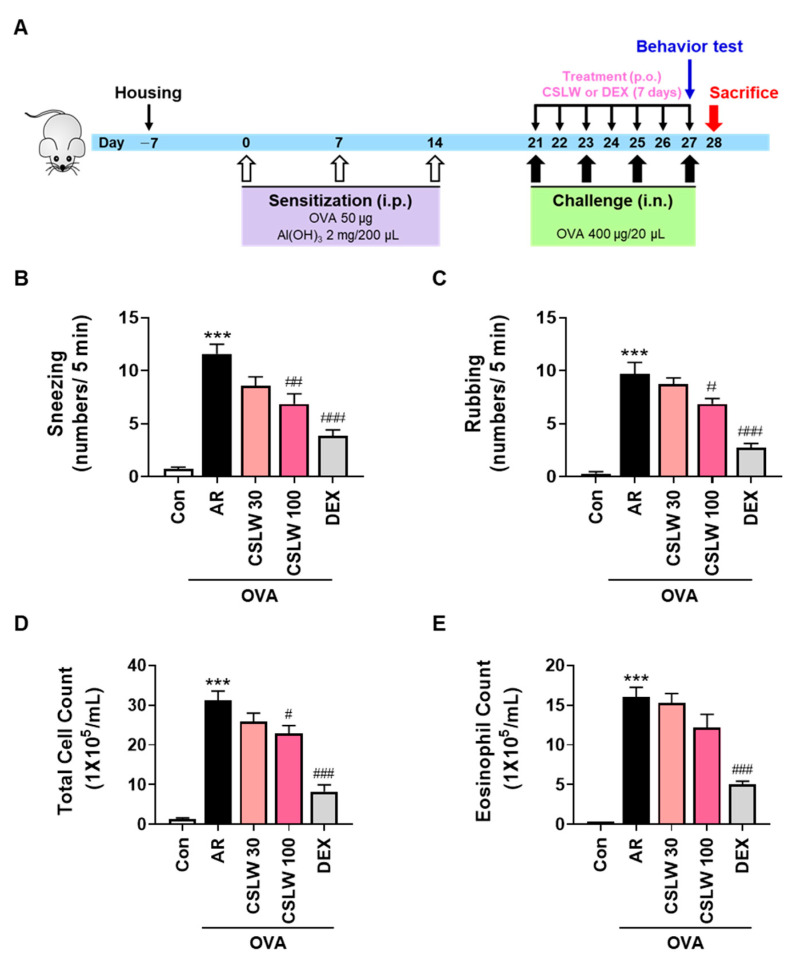
Effects of CSLW on nasal allergy symptoms and NALF parameters in an OVA−induced AR mouse model. (**A**) Experimental protocol for model development and treatment with CSLW. Once daily, the AR mice were orally treated CSLW or DEX, and then sacrificed for analysis 24 h after the final treatment. The number of sneezing (**B**) and rubbing (**C**) behaviors for nasal allergy symptoms were recorded after the last OVA intranasal treatment on day 28. Total cell (**D**) and eosinophil counts (**E**) were counted in the NALF of AR mice with or without CSLW treatment. The values are expressed as the means ± SD (*n* = 7 mice per group). *** *p* < 0.001 compared with the Con (non−sensitized); # *p* < 0.05, ## *p* < 0.01, and ### *p* < 0.001 compared with the AR group. AR, allergic rhinitis; OVA, ovalbumin; CSLW, *Caesalpinia sappan* Linn. heartwood water extract; DEX, dexamethasone; i.p., intraperitoneal injection; p.o., peroral injection; i.n., intranasal injection.

After a week of acclimation (Figure 2A), all mice except for the Con group (non-sensitized) were intraperitoneally administered 50 μg OVA (Sigma-Aldrich, St. Louis, MO, USA) dissolved in aluminum hydroxide (2 mg, Sigma-Aldrich) on day 0, 7, and 14. On day 21, the mice were randomly assigned to one of five groups (n = 5–8 mice per group): group 1, Con group (non-sensitized); group 2, AR group (OVA-sensitized); group 3, CSLW 30 group (OVA-sensitized with CSLW 30 mg/kg/mice); group 4, CSLW 100 group (OVA-sensitized with CSLW 100 mg/kg/mice); group 5, DEX group (OVA-sensitized with dexamethasone 1 mg/kg/mice). From day 21 to 27, CSLW or DEX was orally administered to mice in groups 2–5 once daily. Mice in the Con group received orally administered phosphate-buffered saline (PBS) once daily. The mice were intranasally administered OVA (400 μg) on day 21, 23, 25, and 27; these mice were sacrificed on day 28 (i.e., 24 h after the final oral administration) using alfaxalone (Jurox Pty Ltd., Rutherford, Australia). 

### 2.4. Determination of Nasal Symptoms

Allergic nasal symptoms were evaluated blindly by counting the frequency of sneezing and nasal rubbing behaviors. One hour after the final OVA intranasal challenge, the instances of sneezing and nasal rubbing were recorded for 5 min.

### 2.5. Nasal Lavage Fluid (NALF) Collection and Cell Counting 

After the final challenge, NALF was immediately collected from the sacrificed mice via cannulation from the upper trachea in the nasal cavity direction followed by gentle washing with 10 mL of ice-cold PBS. The total cell numbers in the NALF were counted using a cell counter (Thermo Fisher Scientific, Waltham, MA, USA). Differential eosinophils in each group were centrifuged using a Hanil Cytospin (Seoul, Korea). The slides were stained with Diff-Quik staining reagent (Sysmex, Chuo-ku, Kobe, Japan) according to the manufacturer’s instructions and compared under a light microscope (Olympus Corporation, Tokyo, Japan) at 400× magnification.

### 2.6. Serum Collection and Measurement of Serum OVA-Specific IgE, Histamine, IL-5, and IL-13 Levels

After anesthetization using an intraperitoneal injection of Alfaxan (Jurox Pty Ltd.) on day 28 (refer to Section 2.3), blood samples were collected from the abdominal vein and centrifuged at 1000*× g* for 15 min at 4 °C for serum collection. Serum was measured using the respective enzyme-linked immunosorbent assay (ELISA) with the corresponding kits for OVA-specific IgE (Cayman Chemical, Ann Arbor, MI, USA), histamine (Enzo Life Science, Farmingdale, NY, USA), IL-5 (R&D Systems, Abingdon, UK), and IL-13 (R&D Systems). These experiments were conducted according to the manufacturer’s protocols. 

### 2.7. Histopathological Analysis of Nasal Tissues

After examining the NALF, the mouse heads were removed, fixed in 10% formalin solution (Sigma-Aldrich) for 7 days at 24 °C, decalcified in 0.1 M EDTA buffer (Bio-solution Co. Ltd., Seoul, Korea) for 14 days, and embedded in paraffin. The paraffin-embedded tissue blocks were cut into 5 μm thick sections using a microtome, and the tissues were mounted on slides and stained with hematoxylin and eosin (H&E; Sigma-Aldrich) to analyze the nasal mucosa thickness. A periodic acid Schiff (PAS) staining kit (Sigma-Aldrich) was used to analyze goblet cell hyperplasia, and Giemsa staining solution (Tissue Giemsa stain, BBC Biochemical, Mount Vernon, WA, USA) was used to analyze eosinophil infiltration in the nasal mucosa. Tissue slides were scanned with Pannoramic DESK (3DHISTECH, Budapest, Hungary) digital slide scanners and observed using the Pannoramic CaseViewer software (3DHISTECH).

### 2.8. Immunohistochemical Analysis of Nasal Tissues

The nasal tissues sections of the mice were deparaffinized in xylene, rehydrated, and washed. For immunohistochemical staining, the nasal tissue sections were incubated overnight at 4 °C with rabbit anti-periostin (1:100 dilution; PA5-34641, Thermo Fisher Scientific), mouse anti-MUC5AC (1:1000 dilution; NBP2-15196, Novus Biologicals, Littleton, CO, USA), and mouse anti-4-hydroxynonenal (4-HNE; 1:100 dilution; bs-6313R, Bioss, Beijing, China) primary antibodies. These antibodies were diluted with antibody diluent solution (cat. no. S0809, Dako, Agilent Technologies, Inc., Santa Clara, CA, USA). Slides were then washed and incubated with secondary antibody (cat. no. MP-7801-15, Vector Labs, Burlingame, CA, USA) for 30 min at 24 °C and then stained with 3,3′-diaminobenzidine (DAB) solution. The slides were scanned with Pannoramic DESK (3DHISTECH) digital slide scanners, and the infiltration of eosinophils and the levels of periostin and MUC5AC were observed using the Pannoramic CaseViewer (3DHISTECH).

### 2.9. Cell Culture

HNEpCs were obtained from PromoCell GmbH (PromoCell, Heidelberg, Germany) and the cells were grown in airway epithelial cell growth medium (PromoCell) at 37 °C in a humidified atmosphere of 95% air and 5% CO_2_. Early passage cells (≤6) were used in all experiments.

### 2.10. Cell Viability

HNEpCs were plated in 96-well plates at a density of 1 × 10^4^ cells/well, with incubation at 37 °C for 24 h. Cells were then incubated in airway epithelial cell basal (AECB) culture media (PromoCell) with various doses of CSLW or *N*-Acetyl-l-cysteine (NAC; Sigma-Aldrich) for 24 h. For determination of cell viability, MTS assay kit (Promega, Madison, WI, USA) was used. Briefly, after removing the CSLW- or NAC-treated medium, 100 μL of medium with MTS reagent was added to each well, and the cells were incubated at 37 °C for 4 h. The absorbance at 490 nm was assessed using a BIO-TEK microtiter plate reader (Synergy HT, Winooski, VT, USA). The viability of cells was presented as the relative percentage compared with control cells.

### 2.11. Measurement of Eotaxin-3, Periostin, and MUC5AC Levels

Eotaxin-3, periostin, and MUC5AC levels were measured using the respective ELISA assay kits (R&D Systems), according to the manufacturer’s standard protocol. HNEpCs were plated in 96-well plates at a density of 1 × 10^4^ cells/well for 24 h and then treated in PromoCell AECB media with IL-4/IL-13 (15 ng/mL) in the presence of CSLW or NAC for 24 h. The levels of eotaxin-3, periostin, and MUC5AC secreted into the culture media were measured in culture supernatants using the ELISA kits. The data were normalized to those of the controls.

### 2.12. Immunofluorescence Staining 

HNEpCs were cultured in 12 mm glass-base dishes (Thermo Fisher Scientific) and tested with different doses (1, 3, or 10 μg/mL) of CSLW or 10 mM of NAC after stimulation with IL-4/IL-13 (15 ng/mL) at 37 °C for 1 h. Following the treatment, HNEpCs were washed twice with PBS and then incubated in PromoCell AECB media with 5 μM CellROX orange (Thermo Fisher Scientific) for 30 min at 37 °C. The incubated cells were washed again three times with PBS, and the cell nuclei were stained for an additional 7 min with PBS containing 5 μM DRAQ5 (Cell Signaling Technology, Danvers, MA, USA). Stained cells were analyzed using the FV10i confocal microscope (Olympus).

### 2.13. Determination of Protein Levels

HNEpCs were plated on 6-well plates for 24 h at a density of 2.5 × 10^5^ per well before treatment with IL-4/IL-13 (15 ng/mL) in the presence of CSLW or NAC (positive control for oxidative damage) at 37 °C for 1 h. The stimulated cells were then harvested and lysed with an ice-cold Bio-Rad sample buffer (Hercules, CA, USA), and nuclear extracts were prepared with the NE-PER Nuclear and Cytoplasmic Extraction kit (Thermo Fisher Scientific) according to the manufacturer’s protocol. Next, proteins (25–30 µg) were separated by electrophoresis using Mini-PROTEAN TGX Precast Protein Gels (Bio-Rad, Hercules, CA, USA) and transferred to polyvinylidene difluoride membranes (PVDF, Bio-Rad). The protein-blotted membranes were incubated overnight with gentle agitation at 4 °C with specific primary antibodies against phospho-extracellular signal-regulated kinase (p-ERK)1/2 (1:1000 dilution; cat. no. 9101; Cell Signaling Technology), ERK1/2 (1:1000 dilution; cat. no. 9102; Cell Signaling Technology), Keap1 (1:1000 dilution; cat. no. 8047; Cell Signaling Technology), HO-1 (1:1000 dilution; cat. no. 70081; Cell Signaling Technology), NQO1 (1:1000 dilution; cat. no. 3187; Cell Signaling Technology), SOD1 (1:1000 dilution; cat. no. 2770; Cell Signaling Technology), proliferating cell nuclear antigen (PCNA; 1:1000 dilution; cat. no. 2586; Cell Signaling Technology), β-actin (1:1000 dilution; cat. no. 4970; Cell Signaling Technology), and Nrf2 (1:1000 dilution; cat. no. ab137550; Abcam, Cambridge, UK). The membranes were then washed with Tris-buffered saline containing 0.1% Tween-20 (TBST) and incubated with secondary antibodies (horseradish peroxidase-conjugated anti-IgG) for 1 h at 24 °C. After washing with TBST, the signals on the membranes were detected with EzWestLumiOne-enhanced chemiluminescence solution (Atto Corporation, Tokyo, Japan), and protein detection was performed using the Bio-Rad ChemiDoc Imaging System. The results were quantified with version 1.52a ImageJ software.

### 2.14. Statistical Analysis

The results are expressed as the means ± standard deviation (SD), and all statistical analyses were performed using the GraphPad Prism 7.0 software (GraphPad Software, Inc., La Jolla, CA, USA) by one-way analysis of variance (ANOVA). Comparisons between groups were performed using Tukey’s multiple test to calculate the statistical significance (*p* < 0.05).

## 3. Results

### 3.1. CSLW Alleviates Nasal Allergy Symptoms and Reduces Immune Cell Accumulation of NALF in OVA-Induced AR Mice

We assessed mouse behavior and NALF cell abundance to determine the anti-allergic effect of CSLW on AR nasal symptoms in an OVA-induced mouse model. As shown in Figure 2B,C, the AR group exhibited markedly elevated frequencies of sneezing and nasal rubbing compared with the Con group. In contrast, the CSLW-treated group exhibited attenuation of these behaviors compared with the AR group; this attenuation was significant in the CSLW 100 group. Similarly, the DEX group (positive control) exhibited significantly alleviated allergic nasal symptoms compared with the AR group. In addition, the total cell and eosinophil counts in the NALF were significantly higher in the AR group than in the Con group, whereas these counts in NALF in the CSLW and DEX groups were lower than those in the AR group (Figure 2D,E). These data show that CSLW plays a therapeutic role in AR and alleviates its symptoms.

### 3.2. CSLW Inhibits OVA-Specific IgE, Histamine, and Th2 Inflammatory Cytokine Levels in OVA-Induced AR Mice

**Figure 3:**
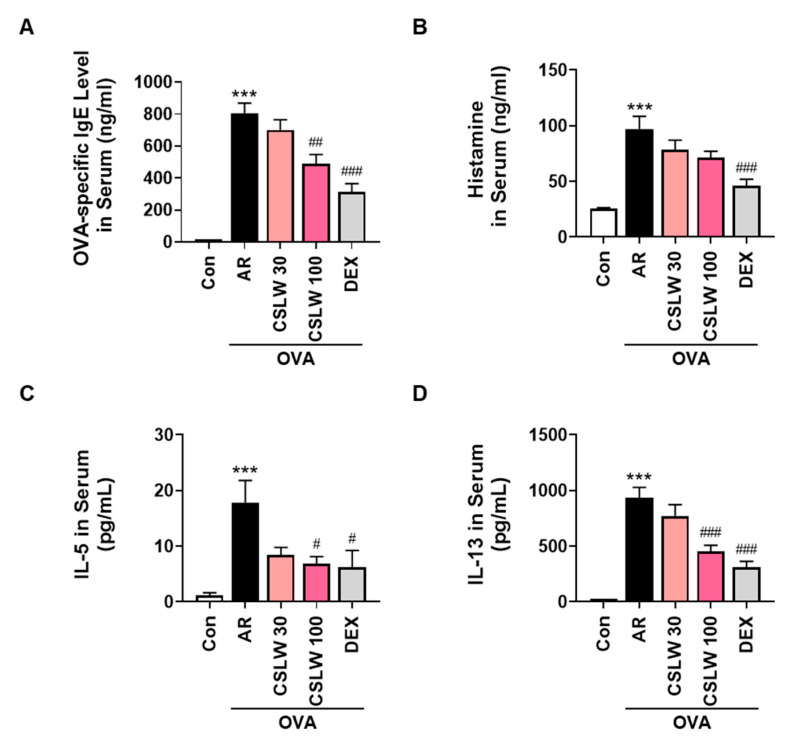
Effect of CSLW on serum levels of inflammatory mediators in an OVA−induced AR mouse model. Serum levels of ovalbumin-specific IgE (**A**), histamine (**B**), IL-5 (**C**), and IL-13 (**D**) were evaluated using ELISA. The values are expressed as the means ± SD (*n* = 6−7 mice per group). *** *p* < 0.001 compared with the Con (non−sensitized) group; # *p* < 0.05, ## *p* < 0.01, and ### *p* < 0.001 compared with the AR group. AR, allergic rhinitis; OVA, ovalbumin; CSLW, *Caesalpinia sappan* Linn. heartwood water extract; DEX, dexamethasone; IL, interleukin.

We examined the serum levels of OVA-specific IgE, histamine, IL-5, and IL-13 to determine whether CSLW improves allergic inflammatory responses in AR mice. The serum levels of OVA-specific IgE, histamine, IL-5, and IL-13 were significantly higher in the AR group than in the Con group (Figure 3). However, the CSLW and DEX groups exhibited much lower levels of these IgE-mediated inflammatory cytokines than the AR group. These results show that CSLW treatment mitigated the allergic responses by inhibiting key allergic inflammation-related mediators, including IgE, histamine, IL-5, and IL-13.

### 3.3. CSLW Inhibits Nasal Mucosa Thickness and Goblet Cell Levels in the Nasal Tissues of OVA-Induced AR Mice

**Figure 4:**
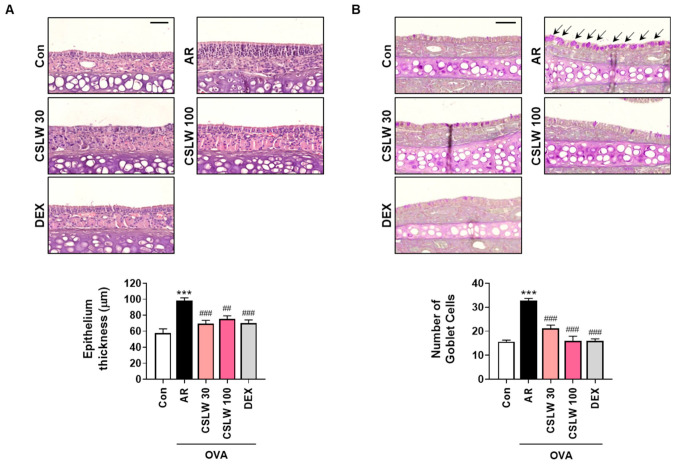
Effect of CSLW on epithelium thickness and goblet cell hyperplasia in nasal tissues. (**A**) General histology, epithelium thickness of nasal mucosa, H&E staining. The bar graph represents the quantification of nasal epithelium thickness (scale bar = 50 µm). (**B**) Goblet cell hyperplasia. PAS staining; the black arrow indicates the goblet cell. Goblet cells are stained purple. Bar graphs represent the quantification of goblet cell counts (scale bar = 50 µm). The values are expressed as the means ± SD (*n* = 6–8 mice per group). *** *p* < 0.001 compared with the Con (non−sensitized) group; ## *p* < 0.01 and ### *p* < 0.001 compared with the AR group. AR, allergic rhinitis; OVA, ovalbumin; CSLW, *Caesalpinia sappan* Linn. heartwood water extract; DEX, dexamethasone.

We performed H&E and PAS staining to investigate whether CSLW affects nasal mucosa histology in AR mice. Nasal epithelium thickness was significantly higher in the AR group than in the Con group because of the accumulation of infiltrating inflammatory cells under the epithelium (Figure 4A). In contrast, epithelial swelling was partially ameliorated after the administration of CSLW and DEX (Figure 4A). In addition, goblet cell hyperplasia, an important feature of airway epithelium inflammation associated with mucus production, was more clearly observed in the nasal mucosa tissue of the AR group than in that of the CSLW and DEX groups (Figure 4B). These results suggest that CSLW regulated AR-related inflammation of the nasal epithelium by controlling the activity of epithelial and goblet cells.

### 3.4. CSLW Inhibits Eosinophil Infiltration, Periostin and MUC5AC Levels, and Oxidative Damage of Nasal Tissue in OVA-Induced AR Mice

**Figure 5:**
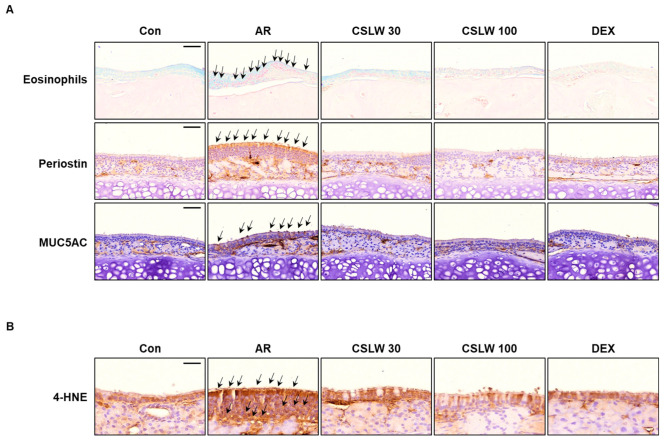
Effect of CSLW on histopathological changes in the nasal tissues. (**A**) Eosinophils stain via Giemsa staining. Infiltration of the eosinophils (stained bright red); the black arrow indicates the eosinophil (scale bar = 50 μm). Immunohistochemical staining for periostin (**A**, middle), MUC5AC (**A**, lower), and 4-HNE (**B**). Periostin (arrows indicate allergic inflammation, scale bar = 50 μm), MUC5AC (arrows indicate mucus production, scale bar = 50 μm), and 4-HNE (arrows indicate oxidative damage, scale bar = 20 μm) are stained brown. AR, allergic rhinitis; OVA, ovalbumin; CSLW, *Caesalpinia sappan* Linn. heartwood water extract; DEX, dexamethasone.

To investigate the anti-inflammatory and anti-allergic effects of CSLW in the AR mouse model, we performed Giemsa and immunohistochemical staining of the nasal epithelium. We initially stained for eosinophils to identify the infiltrating inflammatory cells that affected the thickness of the nasal epithelium because of edema of the nasal tissue in the AR group. Eosinophils were more clearly concentrated in the AR group than in the Con group (Figure 5A). These levels were considerably lower in the CSLW and DEX groups than in the AR group. Next, we evaluated the levels of periostin associated with epithelial thickness and goblet cell hyperplasia in allergic diseases. Immunostaining showed that periostin expression was more widely distributed in the AR group compared with the Con group, and CSLW treatment was associated with reduced periostin levels relative to those in the AR group (Figure 5A). Additionally, immunohistochemical staining showed that MUC5AC was more strongly expressed in the nasal epithelium of the AR group than in that of the Con group. MUC5AC expression in the CSLW group was lower than that in the AR group (Figure 5A). As ROS is excessively generated during AR pathogenesis and inflammatory responses are promoted by oxidative damage, we examined the expression levels of 4-HNE, an oxidative damage marker, in the nasal mucosa tissue of AR mice. The accumulation of 4-HNE was significantly higher in the AR group compared with the Con and CSLW groups (Figure 5B). Consequently, these findings indicate that CSLW improved AR symptoms by regulating eosinophil infiltration and periostin and MUC5AC expression and by inhibiting the oxidative damage associated with allergic inflammation.

### 3.5. CSLW Inhibits Eotaxin-3, MUC5AC, and Periostin Expression by Reducing IL-4/IL-13-Induced ROS Production in HNEpCs

**Figure 6:**
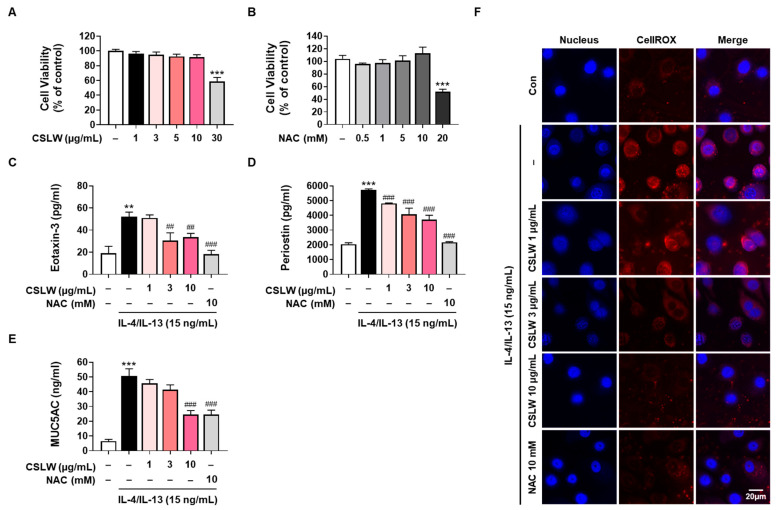
Effect of CSLW on IL−4/IL−13−induced eotaxin-3, periostin, and MUC5AC expression via intracellular ROS production in primary HNEpCs. (**A**,**B**) HNEpCs were treated with the indicated concentrations of CSLW or NAC and incubated for 24 h, and cell viability was evaluated using the MTS assay. Cells were treated with CSLW (1, 3, or 10 μg/mL) or NAC (10 mM) under IL−4/IL−13 (15 ng/mL) −stimulated medium for 24 h (**C**–**E**) or 1 h (**F**). (**C**–**E**) Eotaxin-3, periostin, and MUC5AC levels in the cell culture supernatant were evaluated using an ELISA kit. (**F**) Analysis of intracellular ROS production levels in IL−4/IL−13−stimulated HNEpCs. Cells were stimulated with IL−4/IL−13 (15 ng/mL) under the CSLW or NAC (the latter was used as a positive control) treatment conditions mentioned above and then incubated with the intracellular ROS indicator CellROX and detected via confocal microscopy. The data are presented as the means values ± SD; ** *p* < 0.01 and *** *p* < 0.001 compared with the control cells; ## *p* < 0.01 and ### *p* < 0.001 compared with the IL−4/IL−13−treated cells. CSLW, *Caesalpinia sappan* Linn. heartwood water extract; IL, interleukin; NAC, *N*-acetyl-l-cysteine; ROS, reactive oxygen species.

To determine the activity of nasal epithelial cells under CSLW treatment, we investigated the regulatory effect of CSLW on eosinophil, periostin, and MUC5AC expression and allergic inflammatory response by oxidative damage in HNEpCs. We first analyzed non-toxic concentrations using a cell viability assay to determine the dose-dependent cytotoxic effects of CSLW and NAC on HNEpCs (Figure 6A,B). Next, we performed experiments using IL-4- and IL-13 in HNEpCs and investigated the effects of CSLW by analyzing the secretion of eotaxin-3, periostin, and MUC5AC into the cell culture supernatant. Under IL-4- and IL-13-induced inflammatory conditions in HNEpCs, CSLW was associated with significantly reduced production of eotaxin-3, periostin, and MUC5AC in a dose-dependent manner (Figure 6C–E). A similar inhibitory effect was observed after treatment with NAC. Therefore, we evaluated the effect of CSLW on IL-4- and IL-13-induced ROS generation in HNEpCs to determine whether the synthesis of inflammatory mediators is related to ROS production. As shown in Figure 6F, CSLW treatment reduced IL-4- and IL-13-stimulated intracellular ROS production in a concentration-dependent manner. Collectively, the above results suggest that CSLW exhibited inhibitory effects on nasal inflammation mediated by reduced levels of eotaxin-3, periostin, and MUC5AC via inhibited production of intracellular ROS.

### 3.6. CSLW Inhibits the Production of IL-4/IL-13-Induced Inflammatory Mediators in HNEpCs via Regulation of the ERK-MAPK and Nrf2/HO-1 Signaling Pathways

**Figure 7:**
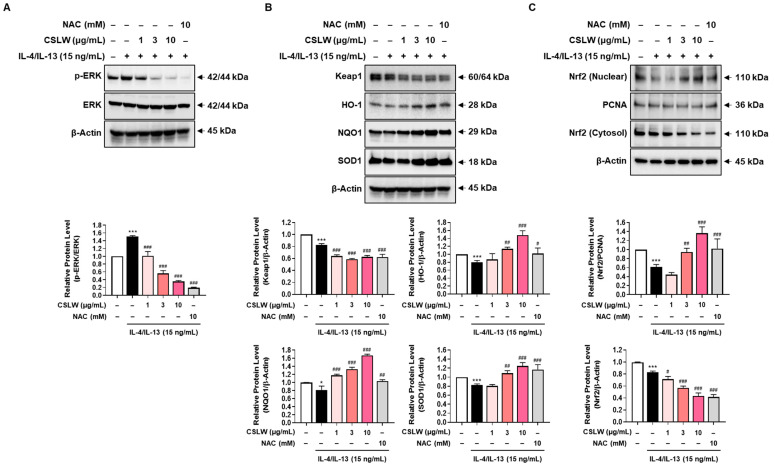
Effect of CSLW on the activation of the EPK−MAPK and Nrf2 signaling pathways in HNEpCs. (A–C) Cells were treated with CSLW (1, 3, and 10 μg/mL) or with NAC (10 mM) along with IL−4/IL−13 (15 ng/mL) for 1 h. (A–C) Expressions of phospho−ERK (p−ERK), ERK, Keap1, HO−1, NQO1, SOD1, Nrf2, and PCNA were evaluated using Western blot analysis. Total β−Actin or PCNA was used to confirm equal sample loading. Images from Western blotting were quantified using ImageJ software. The data are presented as the means values ± SD; * *p* < 0.05 and *** *p* < 0.001 compared with the control cells; # *p* < 0.05, ## *p* < 0.01, and ### *p* < 0.001 compared with the IL-4/IL-13-treated cells. CSLW, *Caesalpinia sappan* Linn. heartwood water extract; HNEpCs, human nasal epithelial cells; MAPK, mitogen-activated protein kinase; Nrf2, nuclear factor erythroid 2−related factor 2.

We first examined MAPK activation using Western blot analysis to identify the underlying molecular mechanisms by which CSLW affects intracellular ROS production. As shown in Figure 7A, IL-4/IL-13 promoted the phosphorylation of ERK-MAPK in HNEpCs, whereas CSLW and NAC treatment inhibited ERK phosphorylation in a dose-dependent manner. Next, we investigated the role of the Keap1/Nrf2 signaling pathway and antioxidant enzymes in the reduction of ROS by CSLW. To this end, we evaluated the effects of CSLW on Nrf2 signaling pathways, including Keap1, NQO1, and HO1, in IL-4/IL-13-stimulated HNEpCs. The expression of the Nrf2 repressor Keap1 was decreased in a concentration-dependent manner after CSLW treatment and showed similar results under NAC treatment (Figure 7B). Conversely, in HNEpCs, IL-4/IL-13 was associated with the decreased expression of HO-1 and NQO1, and the expression of these antioxidants was significantly increased by CSLW treatment (Figure 7B). As seen in Figure 7B, the levels of SOD, a major indicator of oxidative damage, were also downregulated by IL-4/IL-13, and this effect was reversed by CSLW treatment. Meanwhile, the degradation of Keap1 was promoted by CSLW, indicating that Nrf2 was translocated from the cytoplasm to the nucleus. Therefore, we examined the nuclear and cytosolic expression of Nrf2. IL-4/IL-13 stimulation markedly decreased the expression of Nrf2 in the nucleus, which was increased in a dose-dependent manner in the presence of CSLW, whereas it reduced the level of cytosolic Nrf2 (Figure 7C). These findings suggest that CSLW promoted the nuclear translocation of Nrf2. Based on these data, CSLW may induce the expression of Nrf2 and related factors HO-1, NQO1, and SOD1 in HNEpCs, suggesting that CSLW mediates Nrf2-related oxidation remediation in these cells. Collectively, these results indicate that CSLW inhibits the generation of inflammatory mediators (such as eotaxin-3, periostin, and MUC5AC) through ERK-MAPK/ROS- and Nrf2-dependent mechanisms in nasal epithelial cells.

## 4. Discussion

In this study, we investigated the effect of CSLW on AR-related inflammatory responses, focusing on nasal epithelium injury. Our results show that CSLW is associated with the Nrf2 signaling pathway and functions as an AR inhibitor by substantially reducing inflammatory cell and eosinophil infiltration, Th2-related cytokine and inflammatory mediator production, and intracellular ROS production. AR pathogenesis is associated with an imbalance in Th1/Th2 cytokine levels [36,37,38], as exposure to allergens induces IgE-mediated inflammation leading to inflammatory cell infiltration and inflammatory cytokine production [39]. As this cascade of allergic inflammatory processes occurs in the nasal mucosa epithelium, effective treatment for AR must target the nasal epithelium-mediated immune response.

In recent years, several plants have been reported to show potential for the prevention and treatment of AR [37,40]. CSLW has been found to exhibit anti-inflammatory, anti-arthritic, antioxidant, and anti-allergic functions and to be effective against chronic inflammatory diseases [26,27]. Based on these findings, we investigated the underlying mechanisms by which CSLW regulates eosinophils, allergic inflammatory immune cells and mediators, and oxidative damage in AR models.

We found, for the first time, that CSLW treatment could attenuate allergic immune inflammation and ROS production in an OVA-induced AR mouse model. During the AR response, inflammation of the airway and nasal passage occurs because of the infiltration of various inflammatory cells. CSLW administration inhibited associated allergic symptoms (e.g., sneezing and rubbing), along with the proportion of inflammatory cells in the NALF of AR mice. CSLW reduced the production and secretion of OVA-specific IgE, histamine, and Th2 inflammatory cytokines, such as IL-5 and IL-13, in the serum. In addition, CSLW attenuated the thickening of the nasal mucosa and the abundance of goblet cells associated with AR. These findings suggest that CSLW is a strong candidate for AR treatment, playing a therapeutic role in nasal clinical symptoms.

AR is associated with high levels of the eosinophil chemoattractants periostin and mucin, as well as IgE, histamine, and Th2-related inflammatory cytokines in the epithelium of the nasal mucosa [10,11,12,41,42]. The expression of eotaxin-3 and periostin is increased by the stimulation of Th2 inflammatory cytokines such as IL-4 and IL-13 [6,7,8]. In particular, periostin is a major mediator of tissue remodeling under conditions of eosinophilic inflammation and in various inflammatory diseases [9]. In addition, overexpression of MUC5AC, an important gene regulating mucus production in the airways, leads to mucus overproduction and goblet cell hyperplasia, exacerbating the progression of AR [12,13]. CSLW treatment reduced eosinophil abundance in both NALF and nasal tissues and decreased levels of periostin and MUC5AC in nasal tissues. CSLW treatment also reduced the accumulation of 4-HNE, a marker of oxidative damage. Our results demonstrate that CSLW exerts anti-rhinitis effects by inhibiting oxidative damage in inflammatory conditions and reducing the production of allergic immune inflammatory mediators.

As AR is characterized by eosinophilia and the secretion of several inflammatory mediators in the nasal mucosa epithelium, it is necessary to understand the related regulatory mechanisms to improve symptoms. Therefore, we used HNEpCs to investigate the mechanisms underlying the anti-rhinitis activity of CSLW and found that CSLW significantly inhibited the accumulation of eosinophils and upregulation of periostin, MUC5AC, and ROS in IL-4- and IL-13-stimulated HNEpCs in a concentration-dependent manner. ROS are major messengers in the inflammatory response, and disproportionate ROS production leads to the antioxidant system and oxidative tissue damage, eventually promoting inflammatory responses [18,19]. The activation of MAPK pathways, including p38, ERK1/2, and JNK signaling, is strongly associated with airway inflammation [43,44,45,46]. Furthermore, Nrf2 is involved in the transcriptional activation of the gene that encodes the detoxifying enzyme of ROS [3,47,48]. The Nrf2 signaling pathway plays a key role in the regulation of oxidative damage [24,25].

In this study, we showed that CSLW inhibits the activation of ERK-MAPK by functioning as an ROS-targeting antioxidant. We also found that CSLW reduced ROS production through the Keap1/Nrf2 signaling pathways and promoted upregulation of the antioxidant enzymes HO-1, NQO1, and SOD1. Ultimately, these findings showed that CSLW is a promising anti-inflammatory agent for allergic inflammatory responses in IL-4- and IL-13-induced HNEpCs. Our work provides the first evidence of the functions of CSLW in AR and antioxidant activity through the Keap1/Nrf2/HO-1 signaling pathways. However, further studies are required to investigate whether the active compounds of CSLW used in this study can regulate AR by increasing Nrf2 production and promoting the antioxidative pathway. In future studies, we will specifically investigate the antioxidant mechanisms of CSLW’s major components as against the whole extract, to highlight its potential in clinically treating AR with minimal side effects.

## 5. Conclusions

In conclusion, this study demonstrated that CSLW has therapeutic potential for AR treatment through the regulation of Th2 cytokine-related inflammation mediators, eosinophils, goblet cells, and epithelial cells. CSLW improved AR symptoms by significantly attenuating inflammation of the nasal mucosa. First, in OVA-induced AR mice, CSLW reduced the abundance of inflammatory cells in NALF; decreased the thickness and goblet cell hyperplasia/eosinophil infiltration in nasal mucosa; inhibited serum levels of OVA-specific IgE, histamine, IL-5, and IL-13; and decreased eosinophil, periostin, and MUC5AC abundance within the nasal epithelium. Second, in IL-4- and IL-13-induced HNEpCs, CSLW inhibited the production of eotaxin-3, periostin, and MUC5AC by blocking intracellular ROS-mediated ERK-MAPK activity and inducing activation of the Nrf2/HO-1 signaling pathway. Overall, CSLW may be used as an AR therapeutic agent for attenuating inflammation of the nasal mucosa, suggesting its potential as a therapeutic drug for various allergic diseases.

## Figures and Tables

**Table 1:** Constituents identified in CSLW by UPLC-MS/MS.

No.	Rt (min)	Calculated (*m/z*)	Estimated (*m*/*z*)	Error (ppm)	Adduct	Formula	MS2 Fragments (*m*/*z*)	Identification
1	5.26	303.0874	303.0874	−0.0253	[M − H]^−^	C_16_H_16_O_6_	123, 163, 229	Episappanol
2	5.61	303.0874	303.0874	−0.0253	[M − H]^−^	C_16_H_16_O_6_	231	Protosappanin B *
3	6.02	303.0874	303.0874	−0.0253	[M − H]^−^	C_16_H_16_O_6_	229, 257	Sappanol
4	5.7	285.0768	285.0768	−0.0236	[M − H]^−^	C_16_H_14_O_5_	163	Brazilin
5	8.9	285.0768	285.077	−0.3214	[M − H]^−^	C_16_H_14_O_5_	123, 165	3-deoxysappanone B
6	7.1	283.0612	283.0612	0.1723	[M − H]^−^	C_16_H_12_O_5_	240, 173	Brazilein

Rt, retention time. * Compared with the Rt and mass spectrum of reference standards.

## Data Availability

All data is available contained within the article.

## References

[1] Watts A.M., Cripps A.W., West N.P., Cox A.J. (2019). Modulation of allergic inflammation in the nasal mucosa of allergic rhinitis sufferers with topical pharmaceutical agents. Front. Pharmacol..

[2] Watts A.M., West N.P., Smith P.K., Cripps A.W., Cox A.J. (2022). Adult allergic rhinitis sufferers have unique nasal mucosal and peripheral blood immune gene expression profiles: A case-control study. Immun. Inflamm. Dis..

[3] Piao C.H., Fan Y.J., Nguyen T.V., Song C.H., Chai O.H. (2020). Mangiferin alleviates ovalbumin-induced allergic rhinitis via Nrf2/HO-1/NF-kappaB signaling pathways. Int. J. Mol. Sci..

[4] Dong F.L., Tan J., Zheng Y. (2020). Chlorogenic acid alleviates allergic inflammatory responses through regulating Th1/Th2 balance in ovalbumin-induced allergic rhinitis mice. Med. Sci. Monit..

[5] Pawankar R., Mori S., Ozu C., Kimura S. (2011). Overview on the pathomechanisms of allergic rhinitis. Asia Pac. Allergy.

[6] Izuhara K., Nunomura S., Nanri Y., Ogawa M., Ono J., Mitamura Y., Yoshihara T. (2017). Periostin in inflammation and allergy. Cell. Mol. Life Sci..

[7] James A., Janson C., Malinovschi A., Holweg C., Alving K., Ono J., Ohta S., Ek A., Middelveld R., Dahlen B. (2017). Serum periostin relates to type-2 inflammation and lung function in asthma: Data from the large population-based cohort Swedish GA(2)LEN. Allergy.

[8] Ito Y., Al Mubarak R., Roberts N., Correll K., Janssen W., Finigan J., Mishra R., Chu H.W. (2018). IL-13 induces periostin and eotaxin expression in human primary alveolar epithelial cells: Comparison with paired airway epithelial cells. PLoS ONE.

[9] O’Dwyer D.N., Moore B.B. (2017). The role of periostin in lung fibrosis and airway remodeling. Cell. Mol. Life Sci..

[10] Zajkowska M., Mroczko B. (2021). From allergy to cancer-clinical usefulness of eotaxins. Cancers.

[11] Suzaki I., Kawano S., Komiya K., Tanabe T., Akaba T., Asano K., Suzaki H., Izuhara K., Rubin B.K. (2017). Inhibition of IL-13-induced periostin in airway epithelium attenuates cellular protein expression of MUC5AC. Respirology.

[12] Chung Y.W., Cha J., Han S., Chen Y., Gucek M., Cho H.J., Nakahira K., Choi A.M.K., Ryu J.H., Yoon J.H. (2020). Apolipoprotein E and periostin are potential biomarkers of nasal mucosal inflammation. A parallel approach of in vitro and in vivo secretomes. Am. J. Respir. Cell Mol. Biol..

[13] Knoop K.A., Newberry R.D. (2018). Goblet cells: Multifaceted players in immunity at mucosal surfaces. Mucosal Immunol..

[14] Davis J.D., Wypych T.P. (2021). Cellular and functional heterogeneity of the airway epithelium. Mucosal Immunol..

[15] Guo J., Xu S. (2021). Astragaloside IV suppresses histamine-induced inflammatory factors and mucin 5 subtype AC overproduction in nasal epithelial cells via regulation of inflammation-related genes. Bioengineered.

[16] Calven J., Ax E., Radinger M. (2020). The Airway Epithelium-A Central Player in Asthma Pathogenesis. Int. J. Mol. Sci..

[17] Gohy S., Hupin C., Ladjemi M.Z., Hox V., Pilette C. (2020). Key role of the epithelium in chronic upper airways diseases. Clin. Exp. Allergy.

[18] Lim J.O., Song K.H., Lee I.S., Lee S.J., Kim W.I., Pak S.W., Shin I.S., Kim T. (2021). Cimicifugae rhizoma extract attenuates oxidative stress and airway inflammation via the upregulation of Nrf2/HO-1/NQO1 and downregulation of NF-kappaB phosphorylation in ovalbumin-induced asthma. Antioxidants.

[19] Sugiura H., Ichinose M. (2008). Oxidative and nitrative stress in bronchial asthma. Antioxid. Redox Signal..

[20] Lopes R.A., Neves K.B., Tostes R.C., Montezano A.C., Touyz R.M. (2015). Downregulation of nuclear factor erythroid 2-related factor and associated antioxidant genes contributes to redox-sensitive vascular dysfunction in hypertension. Hypertension.

[21] Wasik U., Milkiewicz M., Kempinska-Podhorodecka A., Milkiewicz P. (2017). Protection against oxidative stress mediated by the Nrf2/Keap1 axis is impaired in primary biliary cholangitis. Sci. Rep..

[22] Seo H.Y., Lee S.H., Lee J.H., Hwang J.S., Kim M.K., Jang B.K. (2020). Kahweol activates the Nrf2/HO-1 pathway by decreasing Keap1 expression independently of p62 and autophagy pathways. PLoS ONE.

[23] Li C., Cheng L., Wu H., He P., Zhang Y., Yang Y., Chen J., Chen M. (2018). Activation of the KEAP1NRF2ARE signaling pathway reduces oxidative stress in Hep2 cells. Mol. Med. Rep..

[24] Yi L., Cui J., Wang W., Tang W., Teng F., Zhu X., Qin J., Wuniqiemu T., Sun J., Wei Y. (2020). Formononetin Attenuates Airway In fl ammation and Oxidative Stress in Murine Allergic Asthma. Front. Pharmacol..

[25] Han M., Lee D., Lee S.H., Kim T.H. (2021). Oxidative stress and antioxidant pathway in allergic rhinitis. Antioxidants.

[26] Jung E.G., Han K.I., Hwang S.G., Kwon H.J., Patnaik B.B., Kim Y.H., Han M.D. (2015). Brazilin isolated from Caesalpinia sappan L. inhibits rheumatoid arthritis activity in a type-II collagen induced arthritis mouse model. BMC Complement. Altern. Med..

[27] Mueller M., Weinmann D., Toegel S., Holzer W., Unger F.M., Viernstein H. (2016). Compounds from Caesalpinia sappan with anti-inflammatory properties in macrophages and chondrocytes. Food Funct..

[28] Niu Y., Wang S.F., Li C.Q., Wang J.M., Liu Z.H., Kang W.Y. (2020). Effective compounds from Caesalpinia sappan L. on the tyrosinase in vitro and in vivo. Nat. Prod. Commun.

[29] Baek N.I., Jeon S.G., Ahn E.M., Hahn J.T., Bahn J.H., Jang J.S., Cho S.W., Park J.K., Choi S.Y. (2000). Anticonvulsant compounds from the wood of Caesalpinia sappan L. Arch. Pharm Res..

[30] Nirmal N.P., Rajput M.S., Prasad R.G., Ahmad M. (2015). Brazilin from Caesalpinia sappan heartwood and its pharmacological activities: A review. Asian Pac. J. Trop Med..

[31] Choi D.H., Hwang H.S. (2019). Anti-inflammation activity of brazilin in TNF-α induced human psoriasis dermatitis skin model. Appl. Biol. Chem..

[32] Hwang Y.H., Jang S.A., Kim T., Ha H. (2019). Anti-osteoporotic and anti-adipogenic effects of Rhus chinensis nutgalls in ovariectomized mice fed with a high-fat diet. Planta Med..

[33] Hulme A.N., McNab H., Peggie D.A., Quye A. (2005). Negative ion electrospray mass spectrometry of neoflavonoids. Phytochemistry.

[34] Tong X.Z., Zhu H., Shi Y., Xu H.T., Wang B., Zhao J.H. (2013). An LC/MS/MS method for simultaneous quantitation of two homoisoflavones: Protosappanin B and brazilin with hypoglycemic activity in rat plasma and its application to a comparative pharmacokinetic study in normal and streptozotocin-treated rats. J. Ethnopharmacol..

[35] Yodha A.W.M., Abdillah M., Indalifiany A., Elfahmi E. (2021). Isolation and identification of antioxidant compounds from methanol extract of sappan wood (Caesalpinia sappan). J. Farm. Sains Praktis.

[36] Ren J.J., Yu Z., Yang F.L., Lv D., Hung S., Zhang J., Lin P., Liu S.X., Zhang N., Bachert C. (2015). Effects of Bifidobacterium breve feeding strategy and delivery modes on experimental allergic rhinitis mice. PLoS ONE.

[37] Dong J., Xu O., Wang J., Shan C., Ren X. (2021). Luteolin ameliorates inflammation and Th1/Th2 imbalance via regulating the TLR4/NF-kappaB pathway in allergic rhinitis rats. Immunopharmacol. Immunotoxicol..

[38] Fan Y., Nguyen T.V., Piao C.H., Shin H.S., Song C.H., Chai O.H. (2022). Fructus Amomi extract attenuates nasal inflammation by restoring Th1/Th2 balance and down-regulation of NF-kappaB phosphorylation in OVA-induced allergic rhinitis. Biosci. Rep..

[39] Fan Y., Piao C.H., Hyeon E., Jung S.Y., Eom J.E., Shin H.S., Song C.H., Chai O.H. (2019). Gallic acid alleviates nasal inflammation via activation of Th1 and inhibition of Th2 and Th17 in a mouse model of allergic rhinitis. Int. Immunopharmacol..

[40] Shao Y.Y., Zhou Y.M., Hu M., Li J.Z., Chen C.J., Wang Y.J., Shi X.Y., Wang W.J., Zhang T.T. (2017). The anti-allergic rhinitis effect of traditional chinese medicine of shenqi by regulating mast cell degranulation and Th1/Th2 cytokine balance. Molecules.

[41] Ebihara N., Takahashi K., Takemura H., Akanuma Y., Asano K., Sunagawa M. (2018). Suppressive effect of quercetin on nitric oxide production from nasal epithelial cells in vitro. Evid.-Based Complement. Alternat. Med..

[42] Li Z.P., Zeng M., Deng Y.H., Zhao J.M., Zhou X.X., Trudeau J.B., Goldschmidt E., Moore J.A., Chu H.W., Zhang W.T. (2019). 15-Lipoxygenase 1 in nasal polyps promotes CCL26/eotaxin 3 expression through extracellular signal-regulated kinase activation. J. Allergy Clin. Immun..

[43] Wang C., Choi Y.H., Xian Z., Zheng M., Piao H., Yan G. (2018). Aloperine suppresses allergic airway inflammation through NF-kappaB, MAPK, and Nrf2/HO-1 signaling pathways in mice. Int. Immunopharmacol..

[44] Bai D., Sun T., Lu F., Shen Y., Zhang Y., Zhang B., Yu G., Li H., Hao J. (2022). Eupatilin suppresses OVA-induced asthma by inhibiting NF-kappaB and MAPK and activating Nrf2 signaling pathways in mice. Int. J. Mol. Sci..

[45] Zhang J., Wang X., Vikash V., Ye Q., Wu D., Liu Y., Dong W. (2016). ROS and ROS-mediated cellular signaling. Oxid. Med. Cell. Longev..

[46] Ma Y., Ge A., Zhu W., Liu Y.N., Ji N.F., Zha W.J., Zhang J.X., Zeng X.N., Huang M. (2016). Morin attenuates ovalbumin-induced airway inflammation by modulating oxidative stress-responsive MAPK signaling. Oxid. Med. Cell. Longev..

[47] Nguyen T., Nioi P., Pickett C.B. (2009). The Nrf2-antioxidant response element signaling pathway and its activation by oxidative stress. J. Biol. Chem..

[48] Motohashi H., Yamamoto M. (2004). Nrf2-Keap1 defines a physiologically important stress response mechanism. Trends Mol. Med..

